# Tissue Plasminogen Activator Toxicity after Submacular Hemorrhage Repair

**DOI:** 10.18502/jovr.v16i3.9445

**Published:** 2021-07-29

**Authors:** Yasmin Islam, S. Gibran Khurshid

**Affiliations:** ^1^University of Florida College of Medicine, Department of Ophthalmology, Gainesville, Florida, USA

##  PRESENTATION

**Figure 1:**
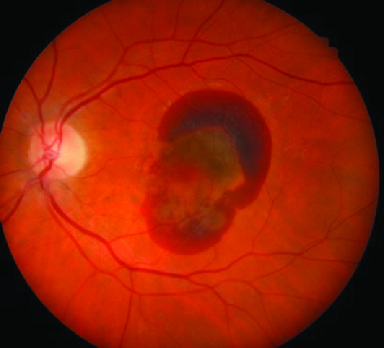
Preoperative color fundus photo demonstrating a large submacular hemorrhage.

**Figure 2:**
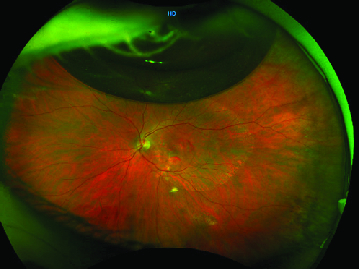
Color fundus photo taken at postoperative week two, demonstrating resolving submacular hemorrhage but new retinal pigment epithelium atrophy extending beyond the area of prior hemorrhage. A C ${}_{3}$ F ${}_{8}$ gas bubble is also present superiorly.

**Figure 3:**
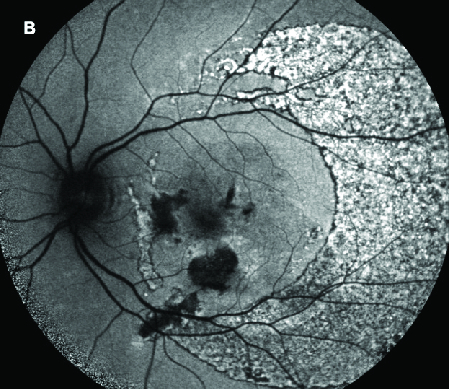
Fundus autofluorescence demonstrating the area of damaged retinal pigment epithelium over the macula that extends temporally at one month postoperatively. There is diffuse hyper-autofluorescence temporal to the macula with areas of hypo-autofluorescence, demonstrating likely retinal pigment epithelium atrophy with areas of hyperplasia.

**Figure 4:**
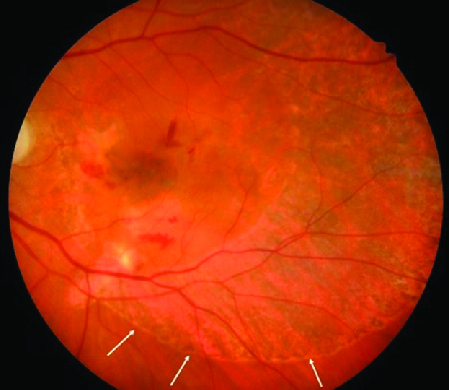
Color fundus photo demonstrating resolved submacular hemorrhage and RPE damage extending from the optic disc to the arcades and the temporal edge of the macula at one month postoperatively. Arrows demonstrate the extent of retinal pigment epithelium damage.

**Figure 5:**
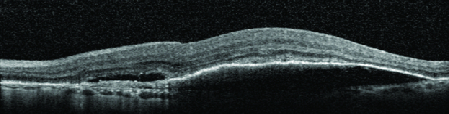
Macular optical coherence tomography at the first postoperative month demonstrating a resolving submacular hemorrhage and retinal pigment epithelium atrophy.

A 76-year-old female presented with a history of non-exudative macular degeneration presented with a submacular hemorrhage (SMH) due to conversion to exudative macular degeneration in the left eye [Figure 1]. Visual acuity had decreased to 20/200. She underwent 23-gauge pars plana vitrectomy, subretinal injection of 0.2 mL of 25 µg/0.1 mL tissue plasminogen activator (tPA) at 2 mm superotemporally to the hemorrhage, and 12% C ${}_{3}$ F ${}_{8}$ gas fill. She postured supine with a 30° forward head tilt for five days to facilitate displacement of the hemorrhage inferiorly. Two weeks after the operation, the hemorrhage had largely cleared, and her vision improved to 20/80, but she developed a crescent-shaped area of retinal pigment epithelium (RPE) atrophy extending beyond the area of tPA injection, with relative sparing of the posterior pole. Figure 2 demonstrates the fundus appearance at two weeks, and Figures 3–5 are the appearance at one month. While SMH can cause retinal toxicity from blood products, this patient's toxicity extended beyond the hemorrhage perimeter and corresponded to the area of tPA infiltration. This likely represents tPA-induced retinal toxicity.

##  DISCUSSION

Subretinal tPA to treat SMH is increasingly used due to its generally excellent outcomes.^[1]^ However, tPA can induce retinal toxicity in a dose dependent manner. Mild toxicity results in localized outer retinal dysfunction in rabbits, while severe toxicity can result in extensive retinal necrosis.^[2]^ Doses ≥ 50 µg have definitively caused toxicity in rabbits and cats when injected intravitreally,^[2,3]^ while studies on toxic doses in humans are limited to case reports. Chen et al described the case of a 49-year-old male who developed tPA-induced RPE changes within five weeks after two 50 µg injections of tPA for SMH into the vitreous cavity. Like our patient, the RPE degeneration extended past the area of SMH, indicating likely tPA toxicity.^[4]^ Hesse et al similarly reported four patients who developed RPE hyperpigmentation after 100 µg doses of intravitreal tPA.^[5]^ However, unlike these prior cases, our patient developed toxicity with a single 50 µg tPA dose subretinally. It is unclear if lower dosages will be safe in the subretinal space, and further studies need to be performed evaluating the safest dosages for subretinal tPA injection.

##  Declaration of Patient Consent

The authors certify that they have obtained all appropriate patient consent forms. In the form the patient has given her consent for her images and other clinical information to be reported in the journal. The patient understand that her name and initial will not be published and due efforts will be made to conceal his identity, but anonymity cannot be guaranteed.

##  Financial Support and Sponsorship

Nil.

##  Conflicts of Interest

There are no conflicts of interest.
